# Response spectrum-based analysis of airborne radar random vibration and multi-point control improvement

**DOI:** 10.1038/s41598-024-56783-y

**Published:** 2024-03-30

**Authors:** Jie Liu, Zezheng Liu, Wanqian Chen, Jing Lv, Zixuan Jiang, Jiahao Pang, Libin Du

**Affiliations:** College of Ocean Science and Engineering, Shandong University of Science and Technology, Qingdao, 266590 China

**Keywords:** Multi-point control, Random vibration, Response spectrum, Synthetic shock response, Virtual excitation, Design, synthesis and processing, Mechanical engineering, Fluid dynamics

## Abstract

During the flight of a UAV (unmanned aerial vehicle), the LiDAR device undergoes random vibrations due to the changing flight attitude and wind speed conditions of the UAV. It is important to control the frequency and amplitude of the vibrations within a reasonable range by means of a damping structure. As the vibrations caused by various factors during flight are random and non-linear, this paper innovates the analysis principle and damping control means for the random vibrations of airborne optoelectronic devices. The response spectrum analysis theory is used to establish the shock response spectrum, and an optimised and improved recursive digital filtering method is used to fit the frequencies of random vibration to the synthetic shock response. Considering the uncertainty of the vibration excitation signal, a virtual excitation method is used for the first time to simulate the random vibration to which the radar may be subjected in the air, and to simplify the calculation steps. The shock plate structure is designed using a multi-point control method to innovate a passive response to the random excitation. Finally, a modal analysis of the synthesised impact response was carried out. It is verified that the first six modal frequencies are controlled within 220 Hz, realising the frequency reduction. The amplitude of the three x, y, and z directions is controlled to within 0.5 mm, thus achieving vibration damping.

## Introduction

UAV-carried LIDAR will encounter the problem of vibration in practical applications. Due to the aerodynamic effect, wind and other factors, the airborne radar will produce various vibrations, and these vibrations lead to changes in the direction and position of the laser beam emission, making the radar data noisy and unstable, affecting the radar performance and detection accuracy. Therefore, solving the vibration problem of UAV-carried LIDAR has always been an important research direction, and the impact of vibration on the performance of LIDAR can be reduced by optimising the structure and materials and increasing the anti-vibration device. This paper analyses and proposes a vibration damping structure design for multi-point control under virtual excitation based on the shock corresponding spectrum in terms of random vibration of airborne lidar.

The study of random vibration can be traced back to 1905, when Einstein introduced the problems related to stochastic analysis in the study of Brownian motion, and then Norbert Weiner proposed the concept of power spectrum of stochastic process in 1930, but the study of stochastic vibration in the real sense did not begin until the 1950s^1–3^. Random vibration is a vibration phenomenon that occurs when an object is subjected to external random forces. In nature and engineering practice, random vibration prevails in various systems, such as structures, mechanical equipment, etc. Random vibration problems are divided into linear random vibration problems and nonlinear random vibration problems, and this paper focuses on nonlinear random vibration problems.

Shock is a physical phenomenon in which mechanical products are subjected to non-repetitive mechanical shocks during use and transport. The shock effect is often an indeterminate, non-periodic transient vibration that induces a vibration response in the structure. Shock response spectra can be used to analyze the response characteristics of structures under these shock loads, to assess the stability and reliability of structures, and to guide engineering design and vibration control. Currently, the shock response spectrum has become an important concept widely used in practical engineering applications. The maximum value of the displacement, velocity or acceleration response produced by a structure during impact action is called the maximum response of the structure^2–5^. The maximum response may occur during the duration of the impact load or after unloading of the impact load, and it depends on the relationship between the intrinsic frequency of the structure and the duration of the impact load.

In the past century, scholars have achieved a wealth of theoretical results in the field of random vibration. However, these theoretical results have encountered intractable problems in engineering practice, such as excessive computation due to vibration irregularities, making it difficult for these theoretical results to be widely used in the engineering community. The reason for this phenomenon is that with the increase of structural degrees of freedom, the traditional method of solving the power spectral density of the random vibration response increases dramatically with the increase of the number of modal vibration patterns used^6,7^. Even if the approximation method is used, i.e. ignoring the correlation terms between modes, the computational volume will increase linearly with the increase in the number of modes used, which leads to a large error, and it is impossible to accurately control the vibration power spectral density of the radar equipment, so that well adapted vibration damping equipment cannot be obtained.

In vibration damping related examples, a well known example is the calculation of nonlinear vibration resistance for multilayer structures considering hysteretic damping mechanisms, using the widely used Bouc-Wen model to build linearised equations boiled down to solving a system of higher order Lyapunov linear equations, or the virtual excitation method to generate a system of lower order narrow-band linear algebraic equations. The latter method is not only simple to solve, and a comparison of the computational volume of a typical example shows that the latter is less than 1/100 of the former, but the computational results are exactly the same. Therefore, the virtual excitation method is widely used by the engineering community to solve various types of nonlinear stochastic vibration problems.

Generally speaking, the experimental method on the structure is highly credible, by applying the actual excitation to make the structure produce the corresponding vibration, and the results are analyzed to solve the actual vibration problems, but this way is difficult to operate and expensive. With the advancement of technology, numerical simulation method is commonly used to solve the vibration simulation in engineering, through the establishment of the dynamic differential equations of the structure, solving the structural dynamics of the theoretical response information, and then achieve the solution of vibration problems, which is characterised by the analysis speed is faster, the design cycle is shorter, and compared with the experimental method, the cost is low, and it can be used to predict vibration tests or analyze the structure that can not be subjected to vibration tests. Based on the numerical simulation method and simulation technology, a corresponding virtual vibration test system can be developed and based on which the vibration analysis of radar structures can be carried out. In this paper, the virtual excitation method is used for the simulation and analysis of vibration, which is not only a simple solution method, but also shows that the calculation results are exactly the same when compared with typical examples. Although the virtual excitation method itself is a linear method, it can still be applied to solve nonlinear random vibration problems.

Thus, this paper proposes a method for modelling and controlling random vibrations on an unmanned airborne radar. As the unmanned airborne radar may be affected by external factors such as wind and airflow during flight, resulting in random vibration on the radar. In order to reduce the impact of such vibration on the radar performance, this paper innovatively proposes the analysis principle and damping control means for the random vibration of airborne optoelectronic equipment, simulates the vibration of the radar structure through virtual excitation calculations, and adopts the principle of multi-point control to install vibration dampers at the key positions of the unmanned airborne radar, so as to achieve the precise control of the system. By monitoring the vibration response and performing feedback control, the vibration of the unmanned airborne radar can be suppressed or reduced. Through the precise control of multi-point control, the frequency and amplitude of vibration can be controlled within a very small range, thus effectively damping the vibration and improving the accuracy of the radar.

## Algorithm principles and simulation analysis

By carrying LIDAR, the drone can scan the location area which is not convenient to detect directly by hand, and efficiently improve the inspection efficiency, during the flight, the carrying LIDAR equipment will be affected by the flight attitude of the drone and wind speed, etc., random vibration will occur, if it cannot adapt to the airborne flight environment, it will fail in the process of carrying out the task, thus affecting the reliability of the equipment, It is therefore essential to keep the frequency and amplitude of vibrations within reasonable limits by means of damping structures^8,9^. The vibration source of the LIDAR comes from various atmospheric phenomena such as changes in the flight conditions of the UAV and wind speed. For this reason, the following method is proposed to design the vibration damping structure, which can effectively improve the detection accuracy of the airborne radar.

### Power spectral density of random vibrations

Vibration isolation measures are designed, analyzed and tested for specific applications and environments and are not universally applicable. Therefore, research work needs to be carried out according to the dynamic performance of the UAS itself and the actual environmental conditions. The vibration signal at the LIDAR installation location is measured in the UAV flight test site and used as an external excitation condition to design the vibration damping device. During the flight of the UAV, the wind and its own vibration factors give random excitation, and the structure will vibrate randomly under the effect of random excitation^10–13^.

Due to the complexity of the natural environment itself, the natural wind shows strong randomness, and the random vibration of the wind field is the main source of vibration, so the simulation design of the wind field should be carried out first. Natural wind can be decomposed into average wind and pulsating wind, the average wind is expressed by the average speed, which does not change with time; the average value of the pulsating wind is zero, and its pulsating component changes with time, and the pulsating response is the response under the action of the pulsating wind, which is caused by the irregular wind of the airframe, and the intensity of the random change of the impact load with time.

The wind speed at the height of z at any time t can be expressed as the sum of the mean wind speed and the pulsating wind speed, and then the mean wind and pulsating wind to which the airborne radar is subjected are defined respectively.

Define $\overline{v }\left(z, t\right)$ as the average wind speed over any time distance τ

1 $$\overline{v }\left(z, t\right)=\overline{v }\left(z\right)+{v}_{f}\left(z, t\right),$$

where $\overline{v }\left(z\right)$ is the average wind speed at height z; ${v}_{f}\left(z, t\right)$ is the pulsating wind speed at height z.

For small, low-altitude UAVs, the 0–1 km altitude is their main activity area, covering the main flight disciplines of take-off, landing and cruise. Since this altitude range belongs to the atmospheric boundary layer, the effects of ground friction and roughness need to be considered, and the description of the distribution of mean wind speed often uses the exponential law formula.

The pattern of variation of mean wind speed along the height can be described by an exponential function:

2 $$\frac{\overline{V} }{\overline{{V }_{s}}}={\left(\frac{Z}{{Z}_{s}}\right)}^{\alpha },$$

where, $\overline{V }$, Z for any point of the average wind speed and height; $\overline{{V }_{s}}$, ${Z}_{s}$ for the standard height of the average wind speed and height, according to the radar flight working altitude of 10 m; $\alpha$ for the roughness coefficient of the ground, the greater the roughness of the ground, the greater α is also usually selected 0.100–0.125.

Steady state calculations are first carried out by setting the wind field velocity inlet conditions to mean wind, as determined by the national loading code, the mean wind velocity profile is described by an exponential model and the wind parameters at a standard height of 10 m. The inlet wind velocity and inlet turbulence intensity are set up in accordance with the following formulas in the code in the range of the gradient wind heights:

3 $$\begin{array}{c}v={v}_{10}{(\frac{z}{10})}^{\alpha }\\ I={I}_{10}{(\frac{z}{10})}^{-\alpha }\end{array}.$$

In the above equation, $v$ and $I$ are the wind speed and turbulence intensity at the calculation height $z$, ${v}_{10}$ and ${I}_{10}$ are the wind speed and turbulence intensity at the standard height of 10 m, and ${v}_{10}$ is taken to be 26.8 m/s.

Based on the above steady-state calculation of the exponential profile-averaged winds at the inlet conditions, and based on the Davenport pulsating wind speed power spectrum and proposed improvements, the harmonic synthesis method is used to simulate the pulsating wind speeds at each height of the inlet boundary, which is combined with the average wind model as the condition of wind field shaking.

The specific expression of the pulsating wind spectrum is as follows:

4 $$\begin{array}{cc}S(n)& =\frac{4k{v}_{10}^{2}{x}^{2}}{n{(1+{x}^{2})}^{4/3}}\\ & x=\frac{1200n}{{v}_{10}}\end{array},$$

where n is the frequency and k is a constant related to the local geomorphology.

**Figure 1:**
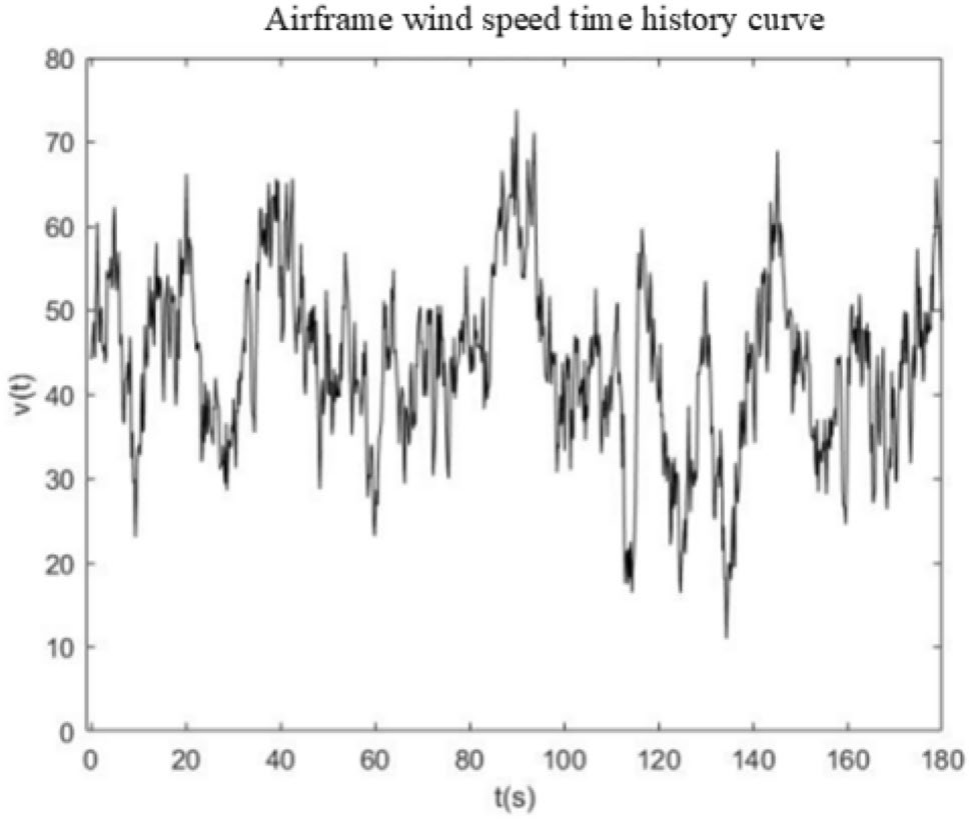
Fuselage wind speed time curve.

In this paper, the simulation of pulsating wind loads on airborne radar is carried out in Matlab based on the Davenport wind spectrum using the harmonic synthesis method of multipoint stochastic process. As shown in Fig. 1. The relevant initial parameters are: (1) fundamental wind speed ${v}_{10}$=26.8 m/s, ground roughness category C, air density is 1.225, ground roughness k = 0.00464; (2) time and frequency parameters. Wind load arrival time t = 0.1 s, total time duration τ = 180 s time step ∆t = 0.2 s, cut-off frequency ${\omega }_{up}$=8πrad/s, frequency range equal fraction N = 500.

**Figure 2:**
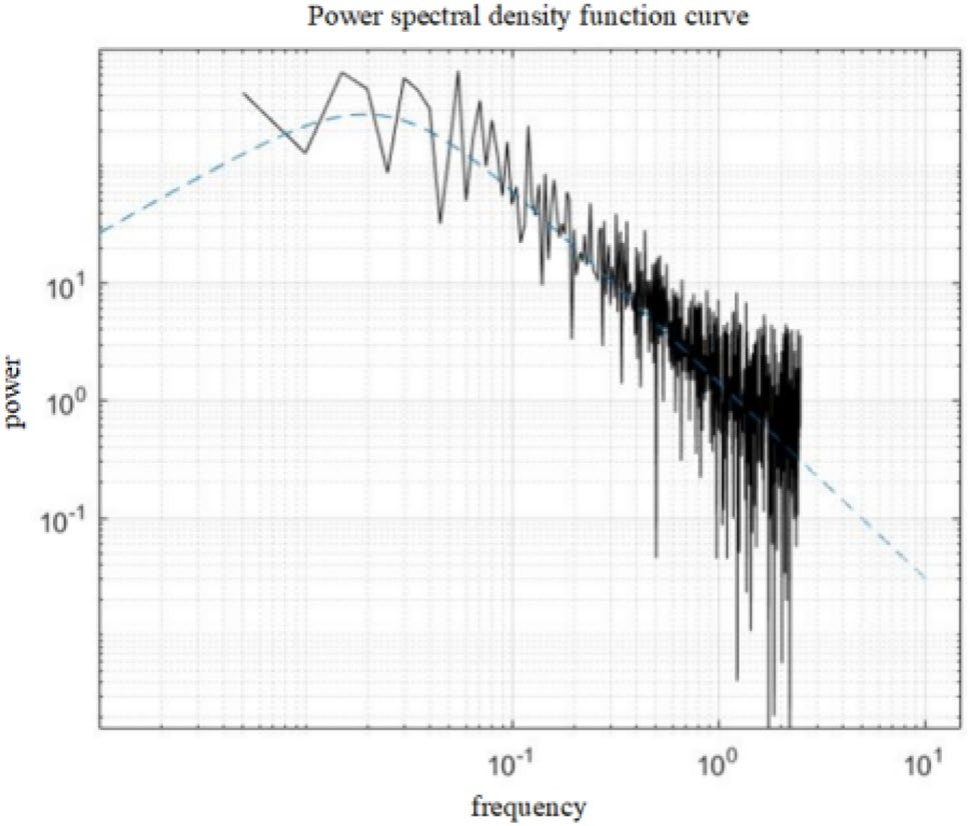
Power spectral density function curve.

The generated wind spectrum is filtered to simulate a pulsating wind field with specific frequency characteristics. Using the generated random pulsations, combined with set peak and mean wind speeds, pulsating wind speed time-range data are generated. Based on the wind speed time-range data, combined with the structural characteristics, the time-range variation of the wind force is calculated and the frequency domain signals are obtained by Fourier Transform (FFT). And the spectrogram is constructed based on the amplitude and phase information of the FFT results, as shown in Fig. 2, to further solve the power spectral density.

The wind field is obtained by computer simulation, and the wind speed time curve of the wind field and the power spectral density of the wind field are derived, which are used as inputs for the subsequent calculation and evaluation of the vibration response.

The statistical information of the random response mainly includes: power spectral density, correlation function, probability density, etc., where the power spectral density and the correlation function are Fourier transform pairs, i.e.

5 $$S\left(\omega \right)=\frac{1}{2\pi }{\int }_{-\infty }^{+\infty }R\left(\tau \right){e}^{-j\omega \tau }d\tau ,$$

where $S(\omega )$ is the power spectral density and $R(\tau )$ is the correlation function. ω is the angular velocity, τ is the time scale.

The goal of this approach is to take into account the dynamic performance of the UAS itself and the actual environmental conditions when designing the damping structure, in order to effectively reduce the impact of excitation on the airborne radar and improve its detection accuracy.

For a linear system with n degrees of freedom, the excitation power spectral density matrix is noted as $[{S}_{xx}]$ and the response power spectral density matrix is $[{S}_{yy}]$. From the above equation, the corresponding frequency response characteristics of the vibration equation in this mode are

6 $${H}_{r}(\omega )=\frac{1}{1-{(\omega /{\omega }_{r})}^{2}+i2{\xi }_{r}\omega /{\omega }_{r}} ,$$

where ${H}_{r}(\omega )$ is the frequency response characteristic, $\omega$ is the angular velocity of power spectrum rotation, ${\omega }_{r}$ is the angular velocity of the correlation function, and ${\xi }_{r}$ is the frequency damping coefficient.

The matrix of correlation functions for the response is defined as follows:

7 $$\begin{array}{c}[{R}_{x}(\tau )]=[{R}_{{x}_{i}{x}_{j}}(\tau )]\\ {R}_{{x}_{i}{x}_{j}}(\tau )=\underset{T\to \infty }{lim}1/T{\int }_{-T/2}^{T/2}{x}_{i}(t){x}_{j}(t+\tau )dt\end{array},$$

where ${R}_{{x}_{i}{x}_{j}}(\tau )$ is the matrix of correlation functions, t is the time to respond once and T is the response frequency period.

This leads to the derivation of the formula for calculating the random vibration power spectrum of a multi-degree-of-freedom linear system. After calculating the information of the power spectrum density function of the system response under the action of a random load, the matrix of the correlation function between the responses can be obtained through the Fourier inverse transform, and then the coherence coefficient, phase difference and other statistical information of the response can be solved, and the working condition of the UAS in this random environment can be calculated from a statistical point of view^14,15^.

The matrix form of the correlation function for the modal coordinates {q(t)} is first analyzed as follows:

8 $$\left[{R}_{q}\left(\tau \right)\right]=\left[\begin{array}{cccc}{R}_{q11}\left(\tau \right)& {R}_{q12}\left(\tau \right)& \cdots & {R}_{q1n}\left(\tau \right)\\ {R}_{q21}\left(\tau \right)& {R}_{q22}\left(\tau \right)& \cdots & {R}_{q2n}\left(\tau \right)\\ \vdots & \vdots & \ddots & \vdots \\ {R}_{qn1}\left(\tau \right)& {R}_{qn2}\left(\tau \right)& \dots & {R}_{qnn}\left(\tau \right)\end{array}\right],$$

where in the correlation matrix $[{R}_{q}(\tau )]$, the diagonal terms are all autocorrelation functions $[{R}_{q}(\tau )]$, rather than cross-correlation functions $\left[{R}_{qij}\left(\tau \right)\right]$ between the responses of the non-diagonal terms.

For the ${R}_{qij}(\tau )$ term, it follows that,

9 $$\begin{array}{cc}& {R}_{qij}(\tau )=E[{q}_{i}(t){q}_{j}(t+\tau )]=\\ & {\int }_{-\infty }^{+\infty }{\int }_{-\infty }^{+\infty }E\left[{Q}_{i}\left(t-{\theta }_{1}\right){Q}_{j}\left(t+\tau -{\theta }_{2}\right)\right]{h}_{i}\left({\theta }_{1}\right){h}_{j}\left({\theta }_{2}\right)d{\theta }_{1}d{\theta }_{2}=\\ & {\int }_{-\infty }^{+\infty }{\int }_{-\infty }^{+\infty }{R}_{Qij}(t+{\theta }_{1}-{\theta }_{2}){h}_{i}({\theta }_{1}){h}_{j}({\theta }_{2})d{\theta }_{1}d{\theta }_{2},\end{array}$$

where $E$ is the desired value sign, ${Q}_{i}$ and ${Q}_{j}$ are two different virtual excitation functions, ${h}_{i}$ and ${h}_{j}$ are the coherence coefficients, ${\theta }_{1}$ and ${\theta }_{2}$ are the excitation parameters meta.

Thus the expression for the correlation matrix $[{R}_{q}(\tau )]$ is

10 $$[{R}_{q}(\tau )]={\int }_{-\infty }^{+\infty }{\int }_{-\infty }^{+\infty }[{R}_{Q}(t+\tau -{\theta }_{2})]\left[h\left({\theta }_{1}\right)\right]\left[h\left({\theta }_{2}\right)\right]d{\theta }_{1}d{\theta }_{2}.$$

Using the transformation relation for the modal coordinates of the equation, it is deduced that

11 $$\begin{array}{*{20}c} {\left[ {R_{y} \left( \tau \right)\left] { = \left[ Y \right]} \right[R_{q} \left( \tau \right)} \right][Y]^{T} } \\ {[R_{Q} \left( \tau \right)\left] = \right[Y]^{T} \left[ {R_{F} \left( \tau \right)} \right]\left[ Y \right]} \\ \end{array}$$

where $Y$ is the virtual excitation generating phase difference.

Substitution gives:

12 $$\left[{R}_{y}\left(\tau \right)\right]=\left[Y\right]{\int }_{-\infty }^{+\infty }{\int }_{-\infty }^{+\infty }\left[h\left({\theta }_{1}\right)\right][Y{]}^{T}{R}_{F}(t+{\theta }_{1}- {\theta }_{2})[Y][h({\theta }_{2})]d{\theta }_{1}d{\theta }_{2}[Y{]}^{T}.$$

Similarly, it is obtained that,

13 $$[{S}_{y}(\omega )]=[Y][H(-i\omega )][Y{]}^{T}[{S}_{F}(\omega )][Y][H(i\omega )][Y{]}^{T}.$$

The resulting random vibration can be equated to an approximate linear system as follows:

14 $$\left[M\right]\left\{\stackrel{\cdot \cdot }{q}\right\}+\left[C\right]\left\{\stackrel{\cdot }{q}\right\}+\left(\left[K\right]+\left[{K}_{e}\right]\right)\left\{q\right\}=\left[F\right],$$

where $\left[{{\text{K}}}_{e}\right]$ is the equivalent stiffness matrix in modal coordinates, $\{{\text{q}}\}$ is the generalised coordinates of the system, $\left[M\right]$ is the system mass matrix, [C] is the damping matrix, [K] is the stiffness matrix [F] is the excitation force matrix.

where the equivalent stiffness matrix in modal coordinates $\left[{{\text{K}}}_{e}\right]$ is expressed as,

15 $$\left[{K}_{e}\right]=[\Phi {]}^{T}\left[K\right]\left[\Phi \right],$$

where [Φ] is the intercepted low-order modal matrix of the system, typically regularised low-order modal information, $\mathrm{\varnothing }$ denotes the offset angle of the vibration point under the condition of $\left[{{\text{K}}}_{e}\right]$ stiffness matrix, and the value of $\left[{{\text{K}}}_{e}\right]$ is determined by the equivalent linearization criterion, which allows the above equation to satisfy the statistically significant minimisation of the error.

The equivalent stochastic vibration of this linear system is computed iteratively, and the most accurate stochastic vibration results are obtained after several iterations of computation.

### Synthetic shock response spectra

**Figure 3:**
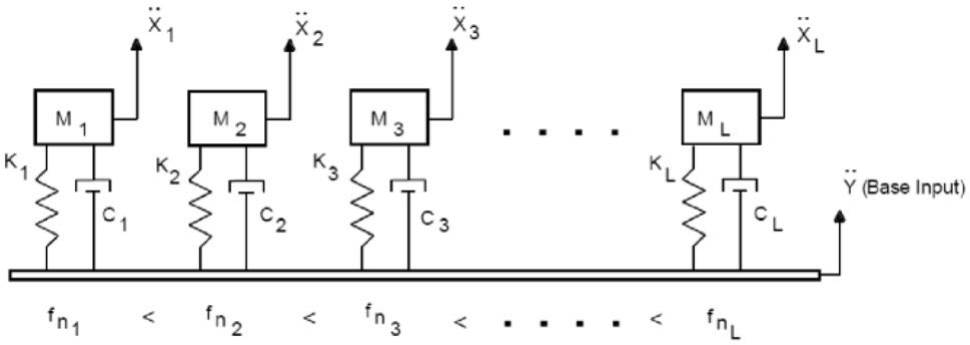
Schematic diagram of the composition of the damping system.

For the vibration of UAVs, impact loading is very important in the design of the structure. Under impact loading, the structure will reach its maximum response in a very short period of time, and the relationship between the maximum response value and the natural frequency is called the impact response spectrum^16^. Impact response spectra can be used to assess the degree of response of a structure at different frequencies and the resistance of the structure to impact loading. For a practical mechanical structure, i.e. a multi-degree-of-freedom system, the impact response spectrum information under impact can be solved by the following method: firstly, the system is decomposed into multiple single-degree-of-freedom spring-mass-damped systems by modal co-ordinate transformation, as shown in the Fig. 3; then, the impact response analysis is carried out for each single-degree-of-freedom system separately to obtain the maximum value of the system response; finally, these values are compared with the corresponding single-degree-of-freedom^17–19^. Finally, these values are combined with the corresponding modal frequencies of the single degree of freedom system to form a series of data points, and a smooth curve is used to connect these data points, so that the impact response spectrum of the whole system under the shock is obtained.

For a single-degree-of-freedom spring-damped system subject to acceleration-based excitation, the differential equations of motion are

16 $$m\ddot{x}+c(\dot{x}-{\dot{x}}_{g})+k(x-{x}_{g})=0,$$

where $x$ is the absolute displacement and ${x}_{g}$ is the base displacement. In the figure ${\ddot{X}}_{1}$, ${\ddot{X}}_{2}$, ${\ddot{X}}_{3}$..${\ddot{X}}_{L}$ are the absolute displacements; M_1_, M_2_, M_3_…M_L_ are the masses of the objects; K_1_, K_2_, K_3_…K_L_ are the spring elasticity coefficients; and C_1_, C_2_, C_3_…C_L_ are the damping coefficients.

Let $\delta =x-{x}_{g}$, representing the relative displacement, then the above equation is deformed to

17 $$m\ddot{\delta }+c\dot{\delta }+k\delta =m{\ddot{x}}_{g}.$$

Namely:

18 $$\ddot{\delta }+2\xi \omega \dot{\delta }+{\omega }^{2}\delta ={\ddot{x}}_{g},$$

where $\xi$ is the frequency response scaling factor.

In this paper, the shock response is first analysed by means of a single-degree-of-freedom system, then the shock response of each single-degree-of-freedom system is multiplied by the corresponding scaling factors, and finally they are superimposed. These scaling factors can be determined by the modal mass and modal shape of each single-degree-of-freedom system, and the resulting superimposed response takes into account the contribution of each single-degree-of-freedom system, thus reflecting the response of the whole system.

Response spectrum analysis is suitable for short-term non-deterministic events such as drone vibration and solves the problem of slow computation of transients in complex structures. The shock response spectrum does not contain phase information between the excitation and the response, so different shock time domain waveforms can have the same shock response spectrum^20–22^. We build a typical base excitation mechanical vibration shock system, i.e. a single degree of freedom system with mass m, elasticity coefficient k and damping c, and use it to construct an absolute acceleration model for the shock response spectrum, where the system input is the base excitation acceleration and the output is the mass block acceleration value.

In this paper, a modified recursive digital filtering method is used to compute the numerical solution of the shock response spectrum, which has the advantage of being fast and accurate. The method is based on the assumption of impulse invariance and approximates the shock input analogue signal model with a rectangular pulse superposition, which is equivalent to the digital recursive filtering method of the single-degree-of-freedom system simulator. The advantage of this method lies in the fact that a digital filter is used for the computation, which avoids complex mathematical calculations in the traditional method and accelerates the computation speed. At the same time, the original shock input signal is approximated by using rectangular pulse superposition, which can better simulate the shock load in the real situation. The calculation formula is,

19 $${x}_{i+1}={P}_{0}{U}_{i+1}+{P}_{1}{U}_{i}+{Q}_{1}{x}_{i}+{Q}_{2}{x}_{i-1},$$

where ${x}_{i}$ is the absolute acceleration response value; ${U}_{i}$ is the base excitation acceleration information; ${P}_{0}, {P}_{1}$, ${Q}_{1}$, ${Q}_{2}$ are the filter coefficients, where,

$${P}_{0}=2\xi \omega \Delta t,{Q}_{1}=2\mathit{exp}\left(-\xi \omega \Delta t\right)\mathit{cos}\left(\omega \Delta t\sqrt{1-{\xi }^{2}}\right){Q}_{2}=-\mathit{exp}\left(-2\xi \omega \Delta t\right),$$

20 $$\begin{array}{c}{P}_{1}=2\xi \omega \Delta texp(-\xi \omega \Delta t)[(1-2{\xi }^{2}/2\xi \sqrt{1-{\xi }^{2}})\\ sin(\omega \Delta t\sqrt{1-{\xi }^{2}})-cos(\omega \Delta t\sqrt{1-{\xi }^{2}})].\end{array}$$

The recursive digital filtering method used is based on a slant table invariant digital model instead of an impulse invariant model, i.e. the generalised slant table function is used instead of the impulse invariant model with $\ddot{u}\left({\text{t}}\right)=\updelta \left({\text{t}}\right)$. The generalised slope table function is $\ddot{u}\left({\text{t}}\right)={\text{A}}\left({\text{t}}-\mathrm{K\Delta t}\right){\text{u}}\left({\text{t}}-\mathrm{K\Delta t}\right)$.

where ${\text{u}}\left({\text{t}}-\mathrm{K\Delta t}\right)$ denotes the unit step function, A denotes the slope of the sloping stage at ${\text{t}}=\mathrm{K\Delta t}$ and $\mathrm{\Delta t}$ denotes the sampling rate of the system. According to the superposition principle a mathematical model of the trapezoidal function approximating the shock input can be obtained, and the method is more accurate compared to the original digital filtering method. Assuming that the sampled value of the acquired input signal u(t) is denoted as ${{\text{U}}}_{{\text{i}}}$, i = 0, 1, 2, …, n. The response of a single degree of freedom system is denoted as x(t), i = 0, 1, 2, …, n. The slope table invariant model recurrence equation is,

21 $${x}_{i}={P}_{0}{U}_{i}+{P}_{1}{U}_{i-1}+{P}_{2}{U}_{i-2}+{q}_{1}{x}_{i-1}+{q}_{2}{x}_{i-2},$$

of which,

22 $${q}_{1}=2exp\left(-{\omega }_{n}\Delta t\xi \right)cos\left({\omega }_{n}\Delta t\sqrt{1-{\xi }^{2}}\right),{q}_{2}=-exp\left(-2{\omega }_{n}\Delta t\xi \right).$$

Absolute acceleration response of the system:

23 $$\begin{array}{c}{P}_{0}=1-exp(-{\omega }_{n}\Delta t\xi )\frac{\mathit{sin}({\omega }_{n}\Delta t\sqrt{1-{\xi }^{2}})}{{\omega }_{n}\Delta t\sqrt{1-{\xi }^{2}}}\\ {P}_{1}=2exp(-{\omega }_{n}\Delta t\xi )[\frac{\mathit{sin}({\omega }_{n}\Delta t\sqrt{1-{\xi }^{2}})}{{\omega }_{n}\Delta t\sqrt{1-{\xi }^{2}}}-cos({\omega }_{n}\Delta t\sqrt{1-{\xi }^{2}})]\\ {P}_{2}=exp(-{\omega }_{n}\Delta t\xi )[exp(-{\omega }_{n}\Delta t\xi )-\frac{\mathit{sin}({\omega }_{n}\Delta t\sqrt{1-{\xi }^{2}})}{{\omega }_{n}\Delta t\sqrt{1-{\xi }^{2}}}]\end{array}.$$

In order to avoid the problem of excessive errors in the calculation results caused by a high sampling rate and insufficient sampling accuracy, the above equation is suitably deformed. Let ${P}_{0}, {P}_{1},{P}_{2}\to 0, {q}_{1}\to 2, {q}_{2}\to -1$.

The following equation can be obtained:

24 $${x}_{i}={P}_{0}{U}_{i}+{P}_{1}{U}_{i-1}+{P}_{2}{U}_{i-2}+{x}_{i-1}+\left({x}_{i-1}-{x}_{i-2}\right)+\left({q}_{1}-2\right){x}_{i-1}+\left({q}_{2}+1\right){x}_{i-2}.$$

The synthetic shock wave expression is obtained as,

25 $$x\left(t\right)=\sum_{i=1}^{k}{A}_{i}{\text{sin}}\left(2\pi f\left(t-{t}_{di}\right)\right){\text{sin}}\left(\frac{\pi \left(t-{t}_{di}\right)}{{T}_{i}}\right).$$

**Figure 4:**
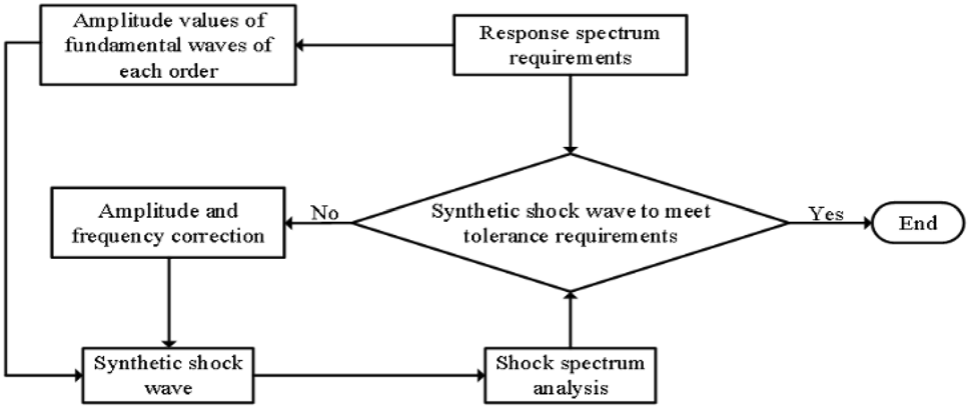
Shock response spectrum flow chart.

The impact response spectrum is derived from the synthesised waveform and determined if the tolerance requirement is met, if not, an iterative correction can be made:

26 $${A}_{imodified}={A}_{i}\times {A}_{gi}/{A}_{\text{resi }},$$

where ${A}_{\text{resi}}$ is the calculated shock response spectrum value, ${A}_{i{\text{mod}}ified}$ is the corrected amplitude, and the shock response spectrum correction process is shown in Fig. 4.

According to the requested unmanned airborne radar shock response spectrum, generate a set of shock waves according to the method of synthesising fundamental waves, solve the shock response spectrum information for them, compare the obtained information with the given shock response spectrum, if it meets the given tolerance requirements, the generated shock signal can be used, if it does not meet the shock spectrum specification, use the above correction process to correct the amplitude of the shock waveform and regenerate the shock signal^23,24^, and so on This is repeated until a shock waveform is generated that meets the shock response spectrum specification.

**Figure 5:**
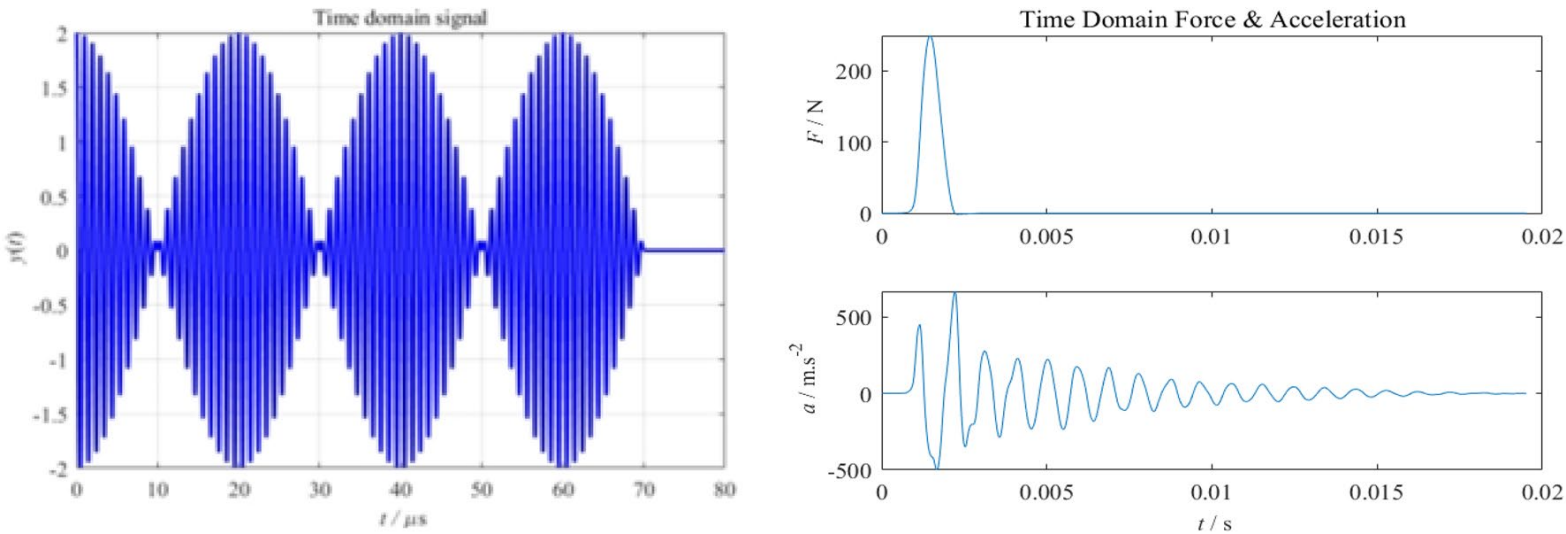
Time domain signal diagram.

The generated time-domain signal map of airborne radar impact response, shown in Fig. 5, can be used to optimise the radar parameters, which is important for the design, commissioning and performance evaluation of the radar system. The LIDAR, which was developed in-house for this paper, has a maximum detection depth of 50 m and a light weight of 20 kg. The LIDAR is subjected to a root mean square acceleration value of less than 8.5 g (rms) over a frequency range of 5 to 2 000 Hz.

**Figure 6:**
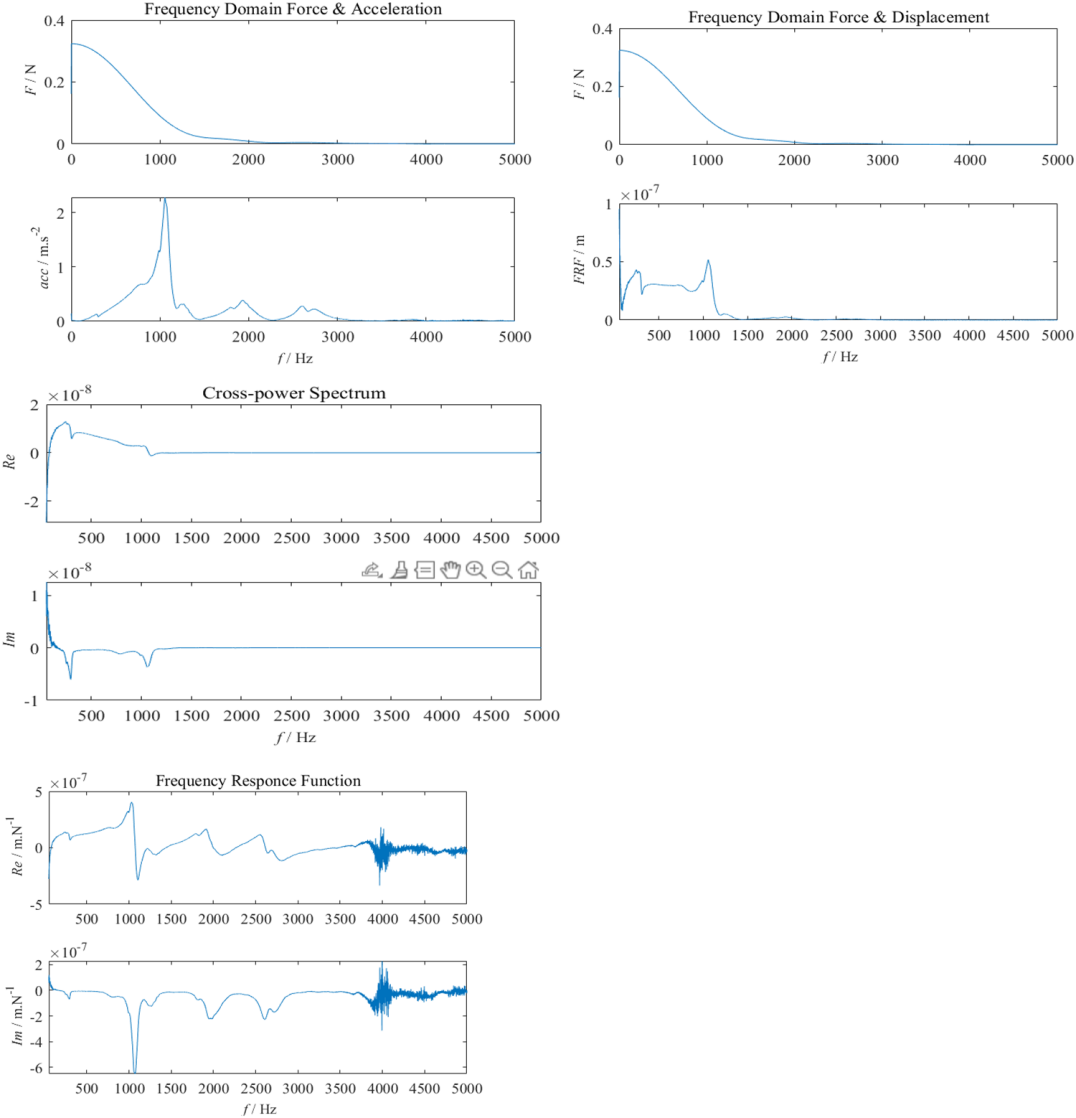
Frequency domain response diagram.

Analysis of the time-domain random vibration response and frequency-domain random vibration response signals obtained from Fig. 6 above shows that: according to the time domain curve, the maximum acceleration amplitude has exceeded the maximum shock acceleration value that the LIDAR can withstand; according to the power spectral density curve, the root mean square acceleration values of the vibration response exceed 8.5 g (rms)^25^, which exceeds the maximum acceleration rms value that the LIDAR can withstand, in addition, the response peak in all directions during the flight of the UAV is generally higher than 250 Hz. In addition, the peak response in all directions during the flight of the UAV is generally higher than 250 Hz. By reasonably designing the vibration isolator structure and using rubber material to design the vibration isolator, the acceleration response of the LIDAR installation location can be effectively reduced.

**Figure 7:**
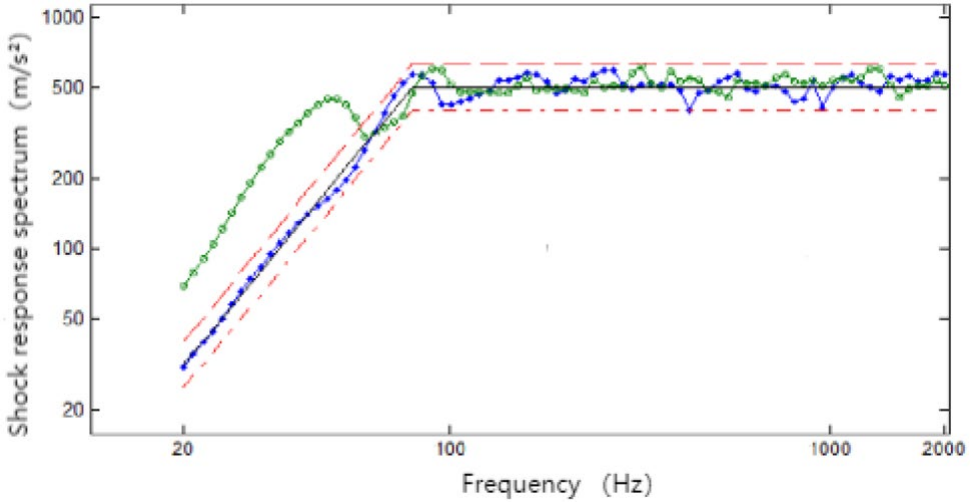
Synthetic shock response spectra.

In the figure, the green line is the vibration spectrum of the external random vibration, and the blue line is the vibration spectrum after the second-order damping. As can be seen from the Fig. 7, although the time delay has a relatively small effect on the shock response spectrum of the synthesised shock signal, relying only on correcting the amplitude of the fundamental waveform may result in the shock response spectrum of the synthesised waveform failing to meet the shock requirements^26^. The use of a random time delay for the fundamental waveform provides good control of the shock response spectrum of the synthesised shock waveform and achieves the specified tolerance requirements.

## Physical design and validation of results

Damper design based on the principles of virtual excitation and multi-point control.

Based on the equivalent non-linear system equations, we have established a non-linear stochastic vibration response analysis method using the stochastic equivalent linearisation technique. By introducing the virtual excitation principle, the method removes the limitation that the original spectral analysis method can only solve for smooth random excitation, and is able to perform random response analysis for smooth and non-smooth random excitation.

The most important feature of the virtual excitation method is that it transforms smooth random vibration analysis into simple harmonic vibration analysis and non-smooth random vibration analysis into deterministic time course analysis, thus simplifying the computational steps significantly while still maintaining theoretical accuracy.

The random signal spectral matrix is then multiplied by the same random phase for the elements in the same column and by different random phases for the elements in different columns in order to ensure correlation between the signals, and then the random signal is solved for each element of the matrix separately^27^. The signal generation reduces the error by using a suitable window function and taking an appropriate correction factor and iterative equalisation, i.e. generating a multi-point random signal.

For the problem of random vibration of a multi-degree-of-freedom system due to multi-point smooth random excitation, the virtual excitation algorithm first needs to decompose the excitation power spectrum matrix $[{{\text{S}}}_{{\text{F}}}(\upomega )]$ as $[{{\text{S}}}_{{\text{F}}}(\upomega )]$=[P]*[P]T. By the nature of matrix multiplication, $[{{\text{S}}}_{{\text{F}}}(\upomega )]$ can also be written as

27 $$\left[{S}_{F}\left(\omega \right)\right]=\sum_{j=1}^{r}{\left\{{\varphi }_{j}\right\}}^{*}{\left\{{\varphi }_{j}\right\}}^{T},j=\mathrm{1,2},3,\dots r,$$

where ${\text{j}}$ is the column vector in matrix [P] and r is the rank of matrix [P].

Thus, the virtual excitation algorithm with multiple points of smooth random excitation is transformed into a virtual excitation algorithm with r single points of smooth random excitation. Therefore, the virtual simple harmonic excitation can be constructed as follows:

28 $$\left\{{F}_{j}\left(t\right)\right\}=\left\{\left\{{\varphi }_{j}\right\}{e}^{i\omega t}\right\}.$$

Then the true response power spectrum of a multipoint smooth random excitation induced random vibration of a multi-degree-of-freedom system is

29 $$\left[{S}_{y}\left(\omega \right)\right]={\sum }_{j=1}^{r}\left[{S}_{{y}_{j}}\left(\omega \right)\right],j=\mathrm{1,2},3,\dots r$$

In practice, UAV vibration dampening platforms are often subjected to multiple points of excitation, and a multi-point excitation vibration analysis of the structure can well simulate the real working environment.

By designing the rubber vibration isolator to a reasonable size, the vibration response of the UAV in flight can be effectively reduced, the LIDAR can be effectively protected and the accuracy of the LIDAR can be effectively stabilised.

**Figure 8:**
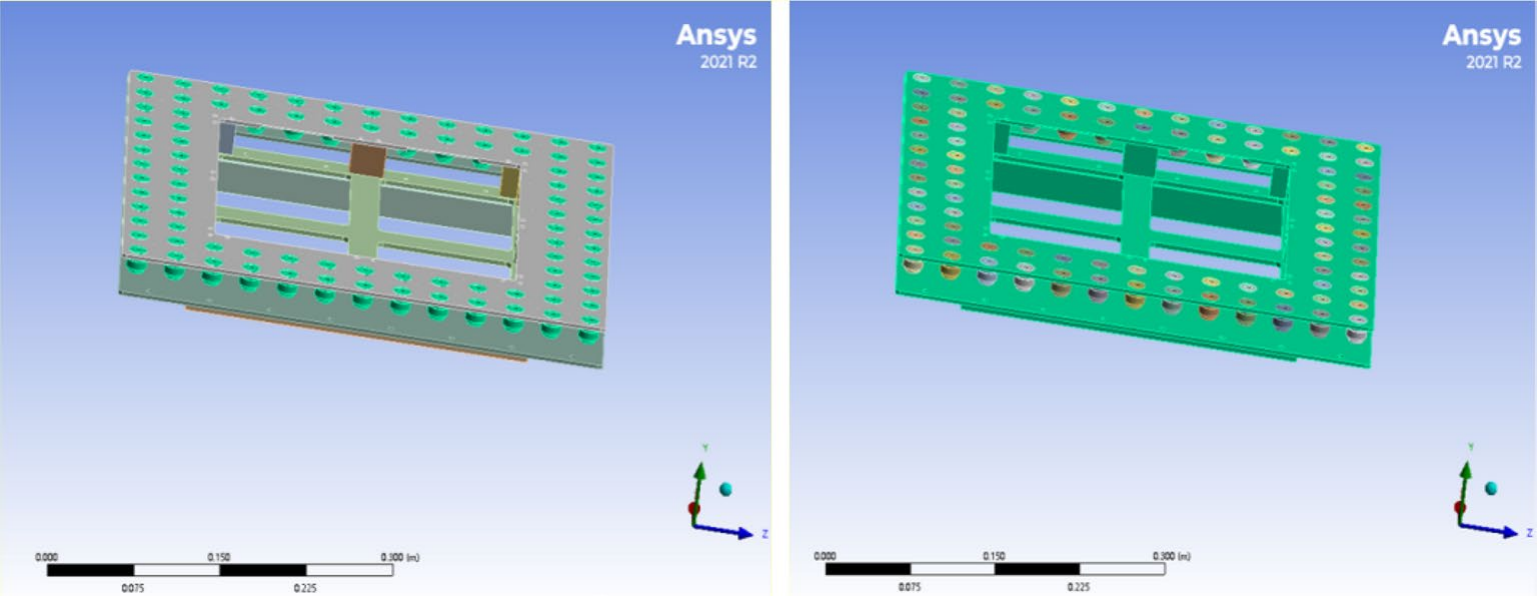
Structure of vibration damping plate.

The vibration isolator used in this paper consists of a vibration plate, an internal spring damping ball, etc., as shown in Fig. 8, which is used to support the weight of the equipment and isolate the vibration transmission to meet the vibration and noise reduction requirements of the unmanned airborne LIDAR part.

The vibration damping plate contains foamed silica gel sheet, which has good elasticity and shock-absorbing properties, and can effectively absorb and disperse vibrations and impacts from the outside. Instead of distributing the foamed silica gel sheets uniformly, the vibration damping plate is provided with multiple control points, and the foamed silica gel sheets are embedded only at the control points. In this paper, the location of the control points is selected by the vibration modal distribution of the structure, and using a stable honeycomb structure, each control point can be regarded as an independent vibration damping unit. When the vibration damping plate is subjected to vibration, the vibration will be dispersed to the various control points, and the foamed silicone sheet will be deformed rapidly to absorb and disperse this energy in order to reduce the vibration response of the structure. Multi-point control of structural vibration is realised to achieve the best damping effect.

The spherical structure of the damping balls, which are built into the two damping plates, allows them to move freely in many directions and to absorb and disperse energy at different angles and positions. This allows the ball to respond well to vibrations and shocks from different directions. In addition, the damping ball can be adjusted by changing its material and geometric parameters to adapt to vibrations and shocks of different frequencies and amplitudes, thus further reducing the transmission of shocks and vibrations.

Compared with the traditional air spring dampers and wire rope dampers, the design solution of built-in damping balls in the two vibration-damping plates can further improve the damping effect by combining the vibration-absorbing properties of the foamed silica gel plates with the vibration-absorbing capabilities of the damping balls, reducing the impacts of vibrations and shocks on the structures and equipments to protect them from normal operation and prolonging their service life.

**Figure 9:**
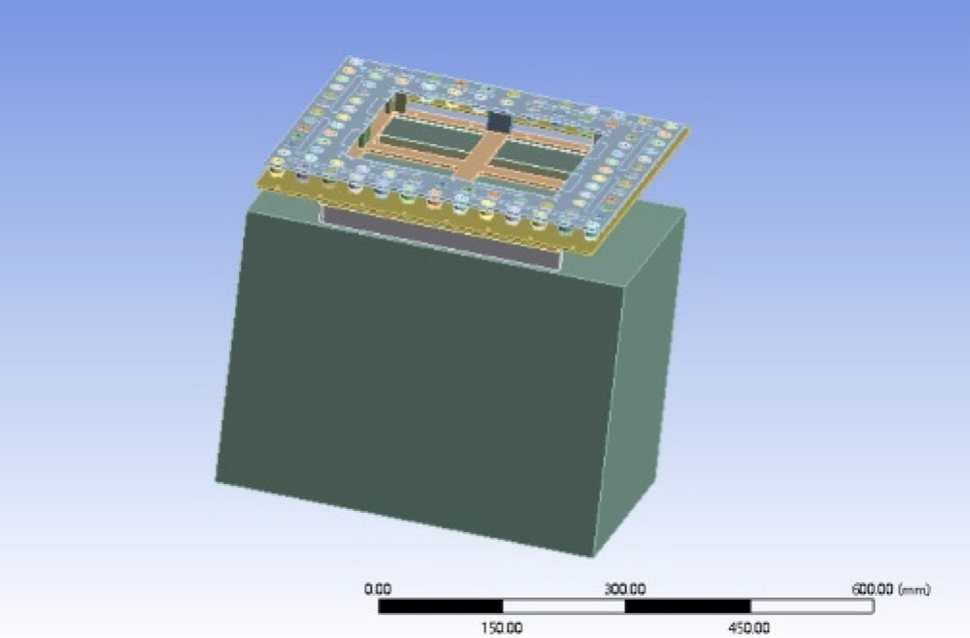
Diagram of parameter condition setting.

As shown in Fig. 9, the three-dimensional model of the unmanned airborne lidar was established. The airborne radar consists of radar shell and vibration damping ball, vibration damping structure also consists of the above components, define the material parameters, aluminium alloy density of 2700 kg per cubic metre, isotropic parameter set Young's modulus 72000000000 Pa and Poisson's ratio of 0.3, the density of rubber is 1200 kg per cubic metre, shear modulus 120,000,000 Pa, Poisson's ratio of 0.49. Define the parameters of deign model to generate the model, all the damping ball is divided into a new part, the rest of the components form new parts. Parameters open deign model to generate model, all the damping ball divided into a new part, the rest of the aluminium alloy parts to form a new part, in order to simplify the model, open mechnical import model to create the material allocation, the damping ball parts to give the rubber material attributes, will be given to the aluminium alloy parts of the aluminium alloy material, mesh division, the mesh size is set to 5 mm, add constraints will be onboard radar Add constraints to set the six pin holes on both sides as fixed constraints, and solve the simulation.

**Figure 10:**
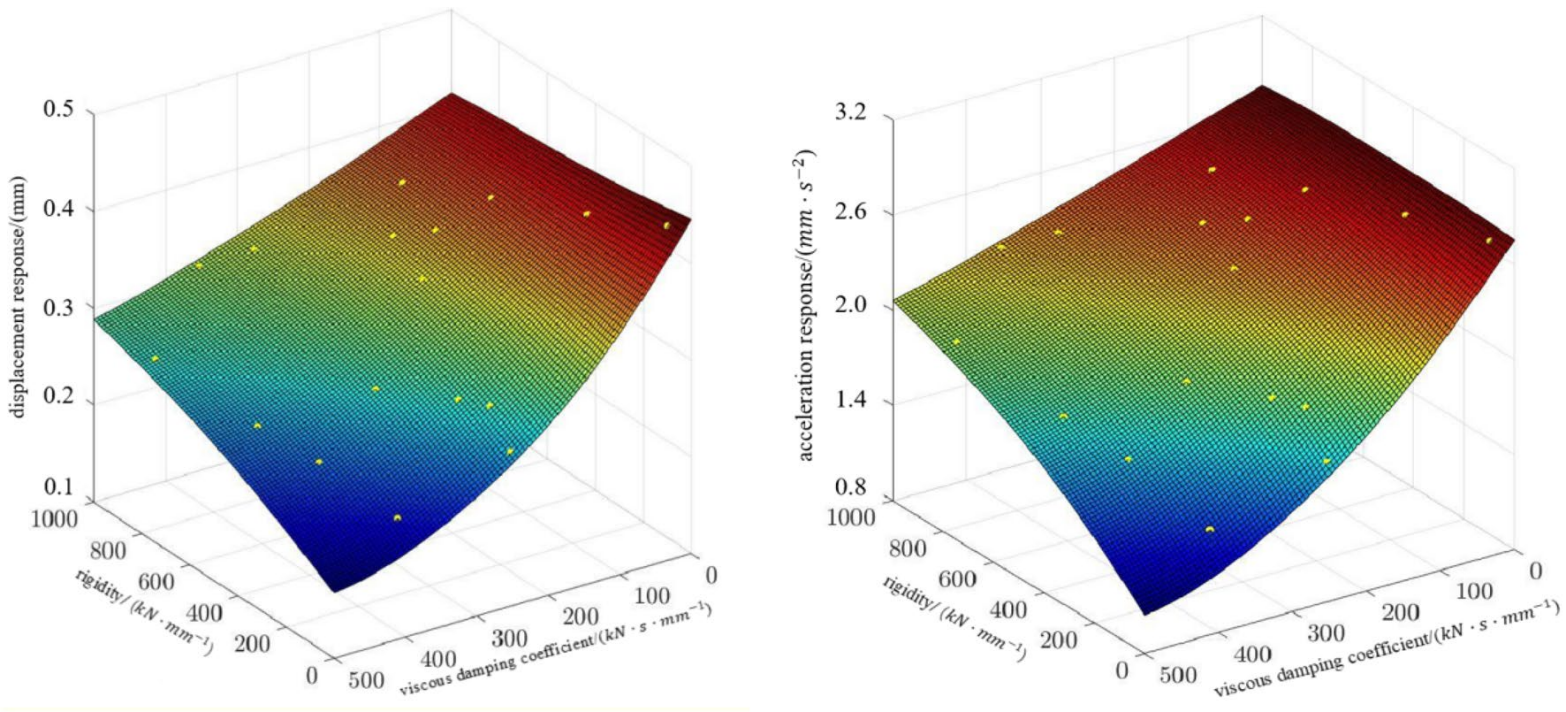
Displacement response and acceleration response.

The vibration response of the radar's damping system was analysed firstly. The second order damped vibration damping system contains elastic and viscous damping. Its displacement response versus stiffness matrix and viscous damping coefficient and acceleration response versus stiffness matrix and viscous damping coefficient are shown in Fig. 10. Adding the random vibration response factor such as vibration wind spectrum and putting it in the second-order damping system simulation, we get the displacement response within 5.3 mm and the acceleration response within 3.2 mm/s^2^, so as to filter out most of the random vibration and transfer it to the radar system with a weak amplitude.

As the rubber material has geometrically large deformation non-linearity in the analysis, the designed vibration isolator is connected to the structure in direct contact and therefore has contact non-linear characteristics^28^. The analysis of the performance of this vibration isolator has special requirements and the commonly used linear analysis methods are not reliable.

**Figure 11:**
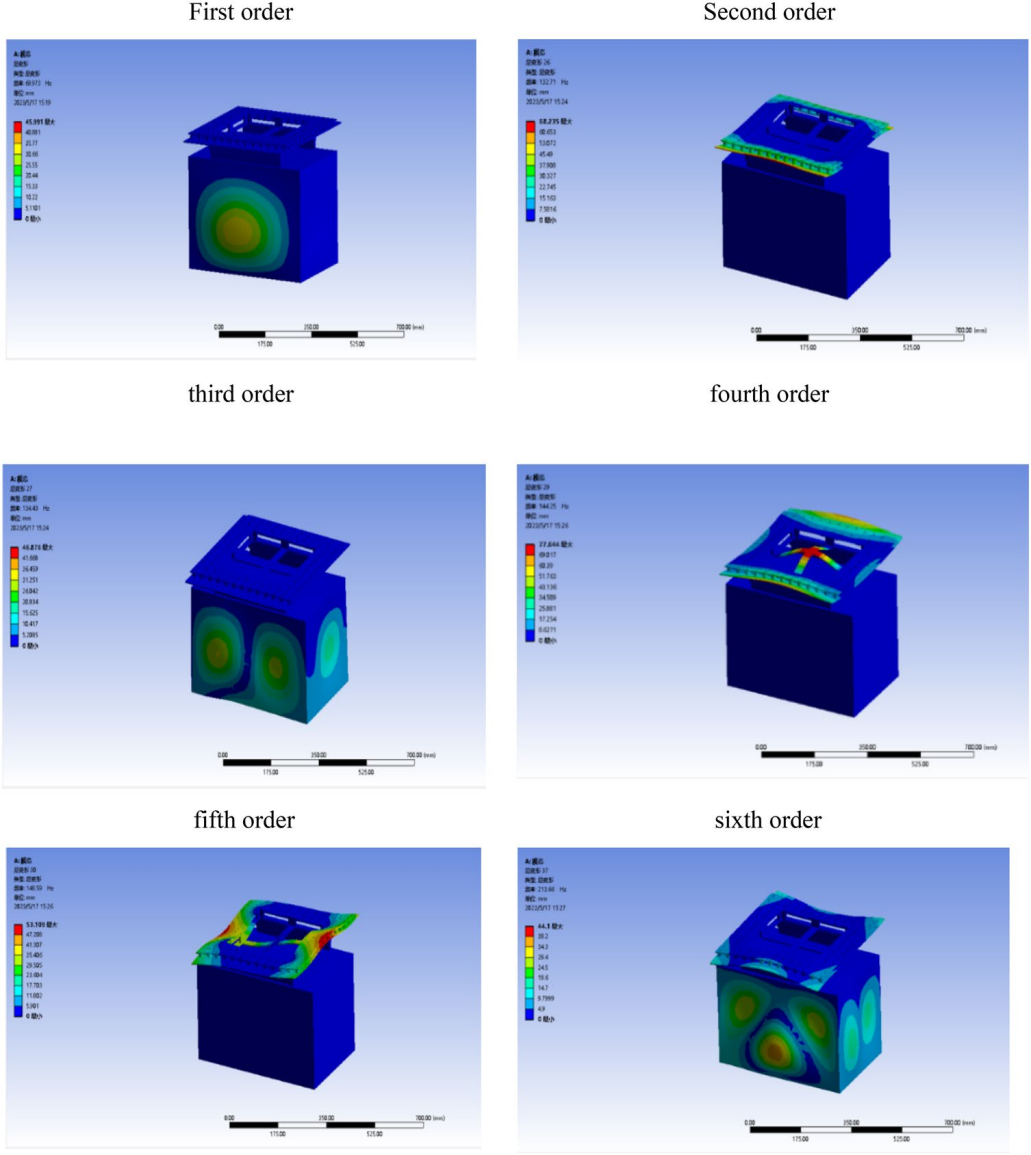
Modal analysis diagram.

Through the simulation analysis, we can get the fundamental frequency of the whole structure after installing the vibration isolator: 69.973 Hz in the first order, 132.71 Hz in the second order, 134.43 Hz in the third order, 144.25 Hz in the fourth order, 143.59 Hz in the fifth order and 213.66 Hz in the sixth order. As shown in Fig. 11, the first-order modes are the excitation frequency of the external force is equal to the intrinsic frequency of the object, the second-order mode is the excitation frequency of the external force is twice the intrinsic frequency of the object, and so on. The first order mode is when the excitation frequency of the external force is equal to the intrinsic frequency of the object, the second order mode is when the excitation frequency of the external force is two times of the intrinsic frequency of the object, the third order mode is when the excitation frequency is two times of the intrinsic frequency of the object, and so on. The whole meets the design requirements of vibration isolation theory. The high-frequency vibration is changed into low-frequency vibration through the damper, so that the displacement and deformation can be controlled within a reasonable range. The acceleration response obtained by finite element analysis shows that the rubber vibration isolator can effectively reduce the vibration response of the LiDAR, and it achieves good vibration isolation effect in three directions, and the root-mean-square acceleration of the vibration analysis response is lower than 8.5grms.

According to the test data provided by the UAV company, the vibration frequency of the multi-rotor UAV is generally concentrated between 550 and 1600 Hz, and the 1st-6th order modes are not in its range.

**Figure 12:**
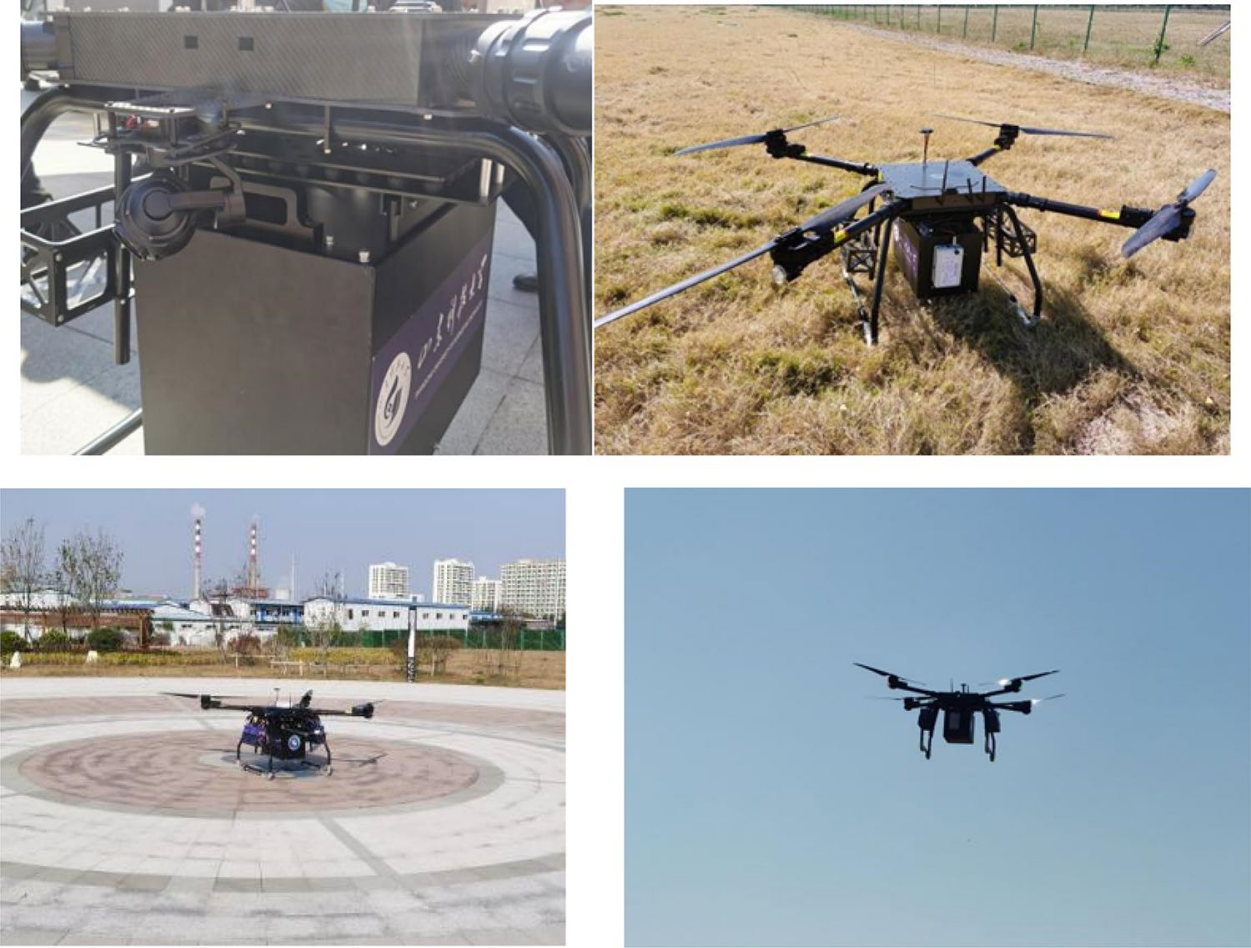
Physical view of the airborne LIDAR.

Based on the installation space of the LIDAR, the size of the structure and the working condition of the LIDAR, it needs to be installed in the form of a suspension to the front position of the UAV mount, so the vibration isolator suitable for the suspension structure is designed, as shown in the Fig. 12.

Multi-point control requires multiple control points in the system or equipment being controlled. The control points are generally selected evenly around the joints, and these control points can be distributed in different locations or parts of the system. Random vibration problems can be well controlled by selecting the right control points. According to whether the number of control points and excitation points is the same, the random vibration multi-point control problem can be divided into square control and rectangular control problems^29^. In practice, the number of control points is usually an integer multiple of the number of excitation points, and this paper focuses on the case where the number of control points is the same as the number of excitation points, i.e. the square control problem.

**Figure 13:**
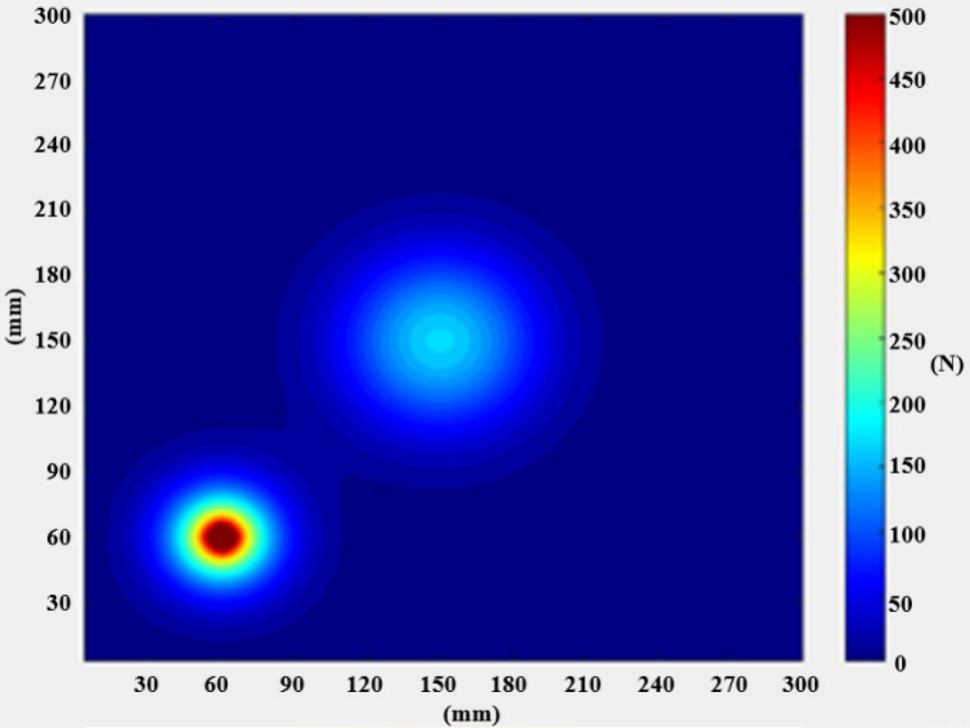
Multi-point control control chart.

In a square control problem, the number of control points is the same as the number of excitation points. This means that each excitation point has a corresponding control point corresponding to it, as shown in Fig. 13. By applying appropriate control forces or control signals on these control points, control of random vibration can be achieved. In this paper, the purpose of reducing the vibration amplitude or suppressing the vibration at a specific frequency can be achieved by using multi-point control algorithms and parameters.

**Figure 14:**
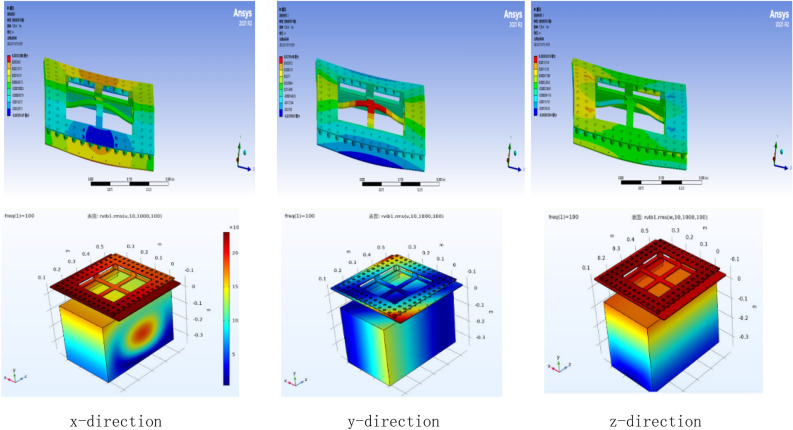
Surface displacement deformation diagram.

The surface displacement and deformation diagram of the radar, as shown in Fig. 14, can be used to determine the shape change of the radar surface according to the displacement, and get the deformation generated by the radar. The vibration damper used in this paper uses rubber material with low modulus of elasticity, which can obtain a lower fundamental frequency and thus improve the vibration damping effect; it also has certain damping characteristics, which can better suppress the resonance response of the low-frequency resonance region. The design of the upper and lower symmetrical damper structure also improves the vibration isolation performance.

**Figure 15:**
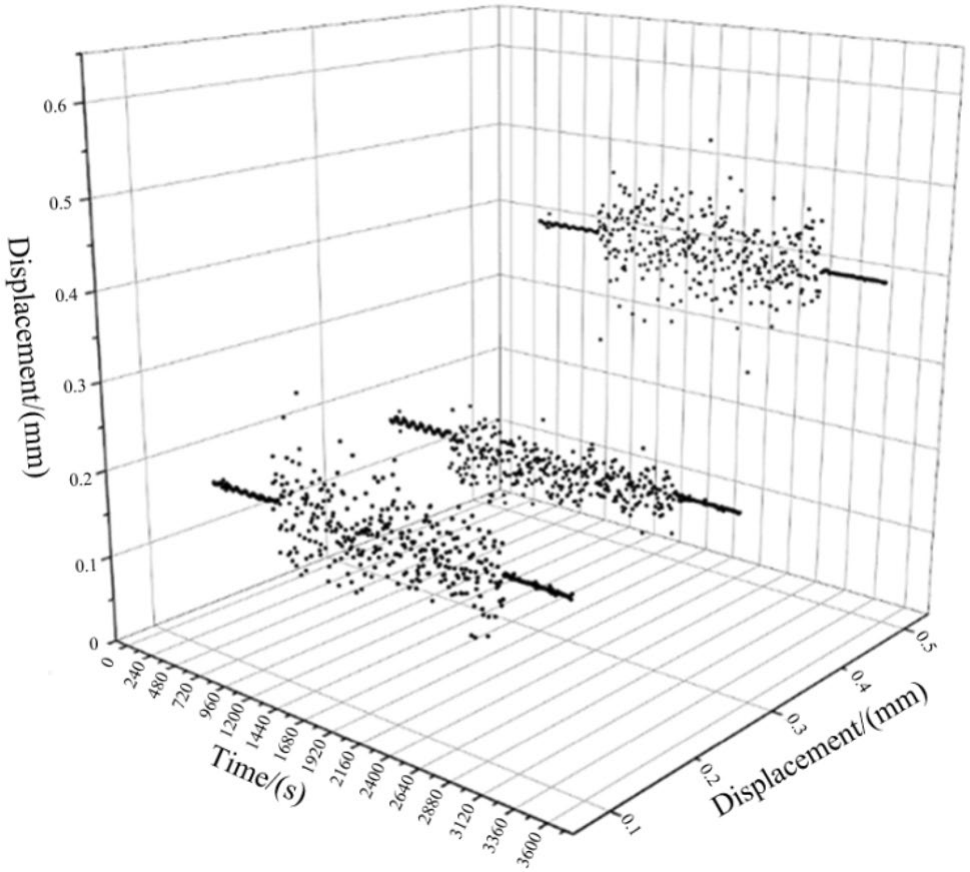
Vibration response results graphs.

From the above vibration response results Fig. 15, it can be seen that, the vibration response through the UAV mount is attenuated by the vibration isolator and then transmitted to the LIDAR with a significant weakening of the vibration response. The root mean square acceleration of the vibration response is below 8 g and the time domain signal amplitude is also significantly weakened. Damping structure to keep radar amplitude within 0.5 mm in x, y, and z directions.

## Results

1. This paper proposes a method for modelling and controlling random vibration on unmanned airborne radar, innovatively puts forward the analysis principle and damping control means of random vibration of airborne optoelectronic equipment, and controls the frequency and amplitude of vibration in a reasonable range by damping structure.
2. This paper adopts the response spectrum analysis theory, establishes the shock response spectrum, and uses the improved recursive digital filtering method to filter and fit the vibration of airborne radar random vibration into a synthetic shock response, which linearizes the vibration frequency and can be displayed visually.
3. The virtual excitation method is applied to solve the problem of uncontrollable random vibration shock sources, while increasing the efficiency of computing. Then the vibration damping plate using multi-point control method, the modal analysis of the synthetic impact response, get its first six orders of modal frequency control within 220 Hz, to achieve frequency reduction requirements.
4. Through the imu data obtained from the experiment, the damping structure can make the radar amplitude in the x y z three directions control within 0.5mm, in line with the design requirements, to achieve the purpose of vibration reduction.

### Supplementary Information

Supplementary Information 1.

Supplementary Information 2.

Supplementary Information 3.

Supplementary Information 4.

Supplementary Information 5.

## Data Availability

All data generated or analyzed in this study are included in this published article and its Supplementary Information file. This includes experimental data, IMU data processed during airborne radar experiments, point cloud data, POSE data, and a full experimental report. -The datasets generated and analyzed in the current study are not publicly available at this time due to a part of the confidentiality involved, but can be obtained from the corresponding author upon reasonable request. Availability of some of the data is limited and these data are used under licence from the current study and are therefore not publicly available.
